# Supplementary material for: DALRD3 encodes a protein mutated in epileptic encephalopathy that targets arginine tRNAs for 3-methylcytosine modification
Source: Nat Commun. 2020 May 19;11:2510. doi: 10.1038/s41467-020-16321-6 (PMC7237682; doi:10.1038/s41467-020-16321-6)
Supplement: Supplementary file 1 — Supplementary Information [file 41467_2020_16321_MOESM1_ESM.pdf]

Supplementary Information:

DALRD3 encodes a protein mutated in epileptic encephalopathy that targets arginine tRNAs for 3-methylcytosine modification

Lentini et al.

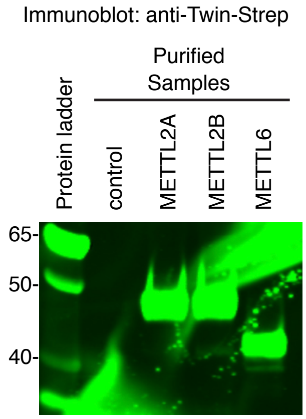

Supplementary Fig. 1. Immunoblot of elutions from streptactin purifications of the indicated proteins. 5% of the total elutions were loaded and probed using antibodies against the twin-Strep tag. The molecular weight ladder is shown on the left in kDa. Blot was repeated three times. Source data are provided as a Source Data file.

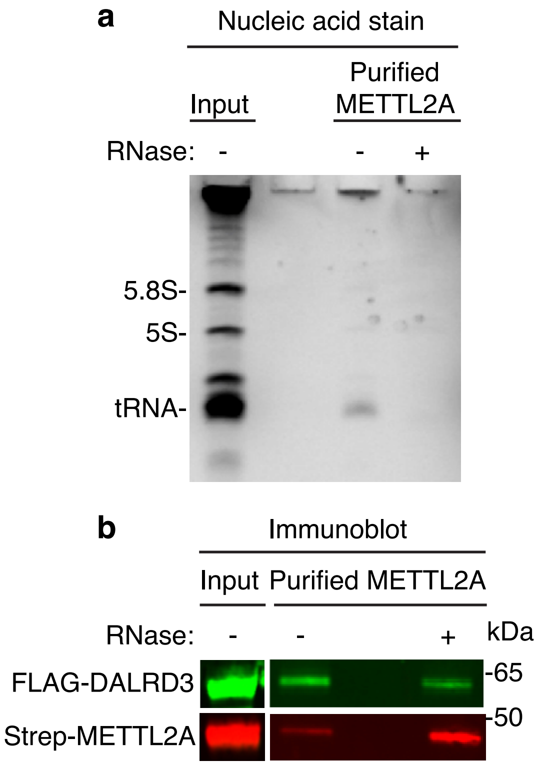

Supplementary Fig. 2. METTL2-DALRD3 complexes are maintained after RNase treatment. (a) Nucleic acid staining of RNA from input extracts and purified Strep-METTL2A samples incubated without or with RNase during purification. The migration pattern of 5.8S rRNA (~150 nt), 5S rRNA (~120 nt) and tRNAs (~70-80 nt) are denoted. (b) Immunoblot of input and purified Strep-METTL2A samples incubated without or with RNase during purification. The experiment was repeated twice with comparable results. Source data are provided as a Source Data file.

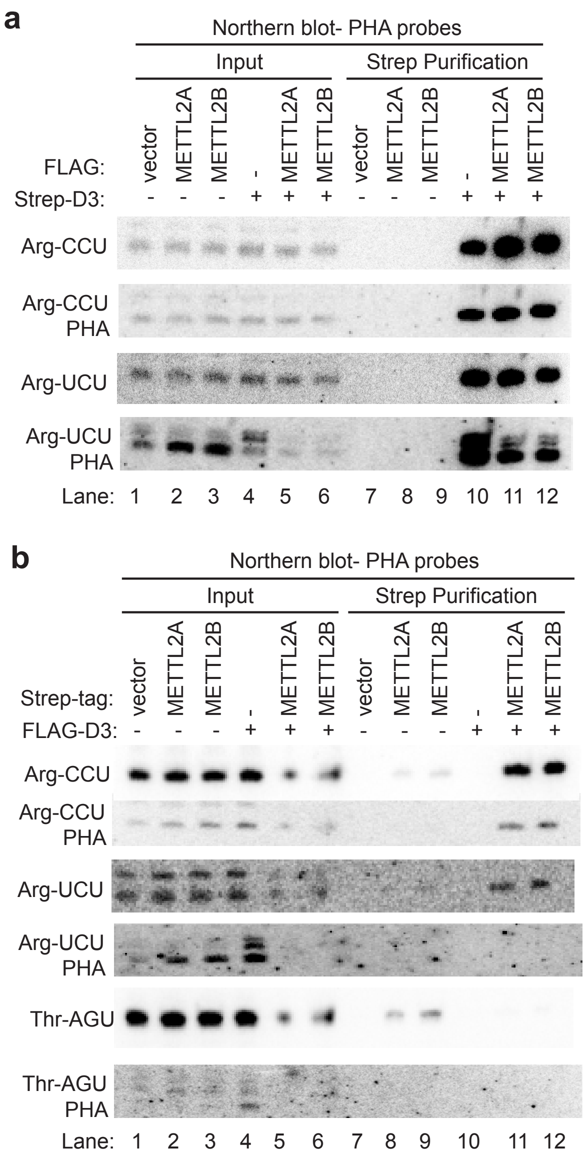

Supplementary Fig. 3. PHA assay for m3C modification of tRNAs from input and METTL2/DALRD3 purifications. (a) Northern blot analysis of purified DALRD3 samples with PHA probes designed to detect m3C at position 32 and a control probe that hybridizes to a different area of the same tRNA. (b) Northern blot analysis of purified METTL2A/B samples with PHA probes designed to detect m3C at position 32 and a control probe that hybridizes to a different area of the same tRNA. The blot was a reprobe of the same blot shown in Figure 3 using the

known lengths of 5.8S rRNA, 5S rRNA and tRNAs. The purification was repeated three times with similar results. Source data are provided as a Source Data file.

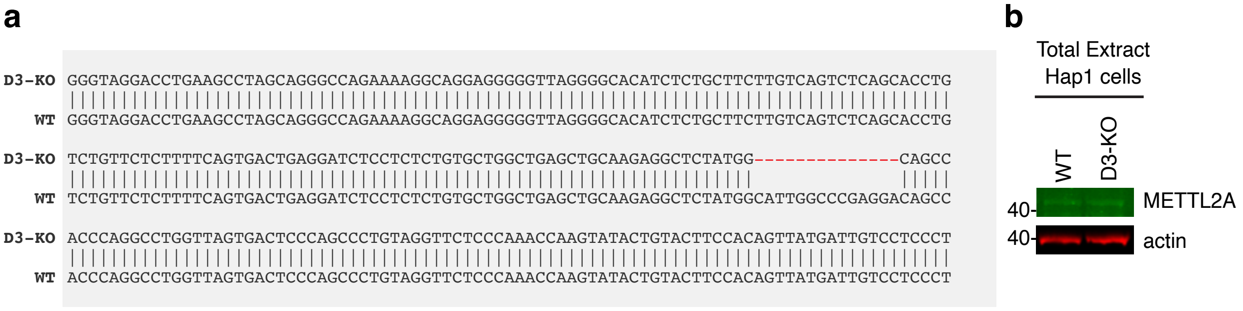

Supplementary Fig. 4. Confirmation of CRISPR-editing of the *DALRD3* locus in HAP1 cells and quantification of METTL2A levels. (a) Sequencing results and alignment of HAP1 WT versus DALRD3-KO cell lines. Note 14-bp deletion that results in a frameshift. (b) Immunoblot of RIPA extracts from Hap1 WT and DALRD3 KO cell lines of the indicated proteins. 2.5% of the total extracts were loaded and probed using antibodies against the METTL2A or actin. The molecular weight ladder is the shown on the left in kDa. Blot was repeated twice with comparable results. Source data are provided as a Source Data file.

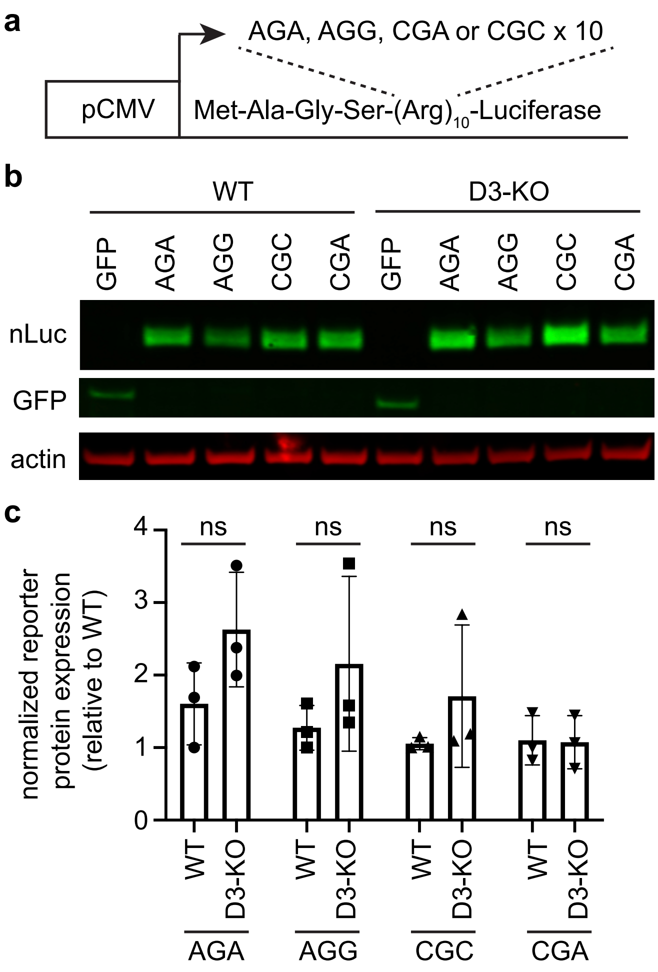

Supplementary Fig. 5. Monitoring arginine codon-dependent translation in WT versus DALRD3-deficient human cells. (a) Schematic of the lentiviral reporter constructs used for generating the human cell lines in (b). The cytomegalovirus (CMV) promoter drives the expression of an mRNA transcript encoding nanoLuciferase (nLuc) fused in frame with the indicated codon run of ten identical arginine codons. (b) Immunoblot of extracts prepared from the stable HAP1 WT or D3-KO cell lines generated using the integrated reporter constructs noted in (a). Green fluorescent protein (GFP) represents a lentiviral construct in which the codon reporter is replaced with the GFP open reading frame as a control. Blot was repeated three times with comparable results. (c) Quantification of codon reporter gene expression. The expression of the luciferase reporter protein (nLuc) or GFP was normalized against endogenous actin and expressed relative to one of the WT cell lines. Error bars indicate the standard deviation from the mean of triplicate samples.

Statistical analysis was performed using a two-tailed, unpaired t-test, if $P < 0.05$ then the difference was considered non-significant (ns). Experiment was performed once. Source data are provided as a Source Data file.

Supplementary Table 1. Oligonucleotides and their sequences.

Primer Name	Sequence (5'→3')
HINDIII-METTL2A F	GAACTAAGCTT ATGGCCGGCTCCTACCCTGAA
NOTI-METTL2A R	CATGAGCGGCCGC TCAGCTGGTGCTGGACAGAAGG
HINDIII-METTL2B F	GAACTAAGCTT ATGGCCGGCTCCTACCCTGAAG
NOTI-METTL2B R	CATGAGCGGCCGCC TATTAGCTGGTGCTGGACAGAAGGGG
HINDIII-DALRD3 F	GAACTAAGCTTATGGCGACCAGGCGCCTTGGGG
NOTI-DALRD3 R	CATGAGCGGCCGCCTATTAAATGTGGCTCAGTGGAGGGAG
Arg-CCU Northern/ Primer Extension probe	GGACTCGAACCCACAaTCCCTGG
Arg-UCU Northern/ Primer Extension probe	TCTCTGCCGGGACTCGAACCCGGA
Arg-CCG Northern probe	GACTCGAACCCTCAaTCTTCT
Arg-ACG Northern probe	GTCGAACCTGGAaTCTTCTGA
Arg-UCG Northern probe	CGACCGCGGCAGGACTCGAACCTGC
Thr-AGU Northern probe/Primer Extension Probe	TTCGAACCCAGGaTCTCCTG
Ser-UGA Northern probe/Primer Extension Probe	AACCTGCGCGGGGAAACCCCAAT
Gln-UUG Northern probe	AGGTCCCACCGAGATTTGAACTCG
U6 snRNANorthern probe	CGTTCCAATTTTAGTATATGTGCTGCCGAAGCGA
tRNA-Arg-CCU template oligo	CGC GGATCC GAAT TAATACGACTCACTATACCCCAGTGGCCTAATGGAtAAGGCATTGGCCTCCTAAGCCAGGGAtTGTGGGTTCGAGTCCCATC TGGGGTGAAGCTTGAACT
tRNA-Arg-CCU R primer	CACCCCAGATGGGACTCGAAC
tRNA-Arg-CCU-C32A template oligo	CGC GGATCC GAAT TAATACGACTCACTATAGCCCCAGTGGCCTAATGGAtAAGGCATTGGCATCCTAAGCCAGGGAtTGTGGGTTCGAGTCCCAT CTGGGGTGAAGCTTGAACT
tRNA-Arg-CCU-U36G template oligo	CGC GGATCC GAAT TAATACGACTCACTATAGCCCCAGTGGCCTAATGGAtAAGGCATTGGCCTCCGAAGCCAGGGAtTGTGGGTTCGAGTCCCAT CTGGGGTGAAGCTTGAACT

tRNA-Arg-CCU-A37G template oligo	CGC GGATCC GAAT TAATACGACTCACTATAGCCCCAGTGGCCTAATGGAtAAGGCATTGGCCTCCTGAGCCAGGGAtTGTGGGTTCGAGTCCCATCTGGGGTGAAGCTTGAACT
Arg-CCU PHA probe	GCTTAGGAGGCCAATG
Arg-UCU PHA probe	GAATTAGAAGTCCAATGCG
R primer DALRD3 ΔDALR	CATGAGCGGCCGCcta tta AAAGGTGCCACTCTTTGTGCC
F primer ΔN-TERM-DALRD3	GAACT AAGCTTATG CTC TCT GTG CTG GCT GAG CT
10xAGA-nLuc gene block	AAGCTTGCCACCATGGCCGGATCCAGAAGAAGAAGAAGAAGAAGAAGAAGAGGTACCGGTGGTGGTGGTTCTGGTGGTGGTGGTTCTGTCTTCACACTCGAAGATTTCGTTGGGGACTGGCGACAGACAGCCGGCTACAACCTGGACCAAGTCCTTGAACAGGGAGGTGTGTCCAGTTTGTTTCAGAATCTCGGGGTGTCCGTAACTCCGATCCAAAGGATTGTCCTGAGCGGTGAAAATGGGCTGAAGATCGACATCCATGTCATCATCCCGTATGAAGGTCTGAGCGGCGACCAAATGGGCCAGATCGAAAAAATTTTTAAGGTGGTGTACCCTGTGGATGATCATCACTTTAAGGTGATCCTGCACTATGGCACACTGGTAATCGACGGGGTTACGCCGAACATGATCGACTATTTCGGACGGCCGTATGAAGGCATCGCCGTGTTCGACGGCAAAAAGATCACTGTAACAGGGACCCTGTGGAACGGCAACAAAATTATCGACGAGCGCCTGATCAACCCCGACGGCTCCCTGCTGTTCCGAGTAACCATCAACGGAGTGACCGGCTGGCGGCTGTGCGAACGCATTCTGGCGGACTACAAAGACCATGACGGTGATTATAAAGATCATGACATCGATTACAAGGATGACGATGACAAGTAAGAATTC
10xAGG-TOP	GATCC AGGAGGAGGAGGAGGAGGAGGAGGAGG GGTAC
10xAGG-BOT	CCCTCCTCCTCCTCCTCCTCCTCCTCCTG
10xCGA-TOP	GATCC CGACGACGACGACGACGACGACGACGA GGTAC
10xCGA-BOT	CTCGTCGTCGTCGTCGTCGTCGTCGTCGG
10xCGC-TOP	GATCC CGCCGCCGCCGCCGCCGCCGCCGCCGC GGTAC
10xCGC-BOT	CGCGGCGGCGGCGGCGGCGGCGGCGGCGG
