# Supplementary material for: DALRD3 encodes a protein mutated in epileptic encephalopathy that targets arginine tRNAs for 3-methylcytosine modification
Source: Nat Commun. 2020 May 19;11:2510. doi: 10.1038/s41467-020-16321-6 (PMC7237682; doi:10.1038/s41467-020-16321-6)
Supplement: Supplementary file 3 — Reporting Summary [file 41467_2020_16321_MOESM3_ESM.pdf]

nature research

Corresponding author(s): Dragony Fu

Last updated by author(s): Mar 23, 2020

Reporting Summary

Nature Research wishes to improve the reproducibility of the work that we publish. This form provides structure for consistency and transparency in reporting. For further information on Nature Research policies, see Authors & Referees and the Editorial Policy Checklist.

Statistics

For all statistical analyses, confirm that the following items are present in the figure legend, table legend, main text, or Methods section.

n/a	Confirmed	
☐	☒	The exact sample size (n) for each experimental group/condition, given as a discrete number and unit of measurement
☐	☒	A statement on whether measurements were taken from distinct samples or whether the same sample was measured repeatedly
☐	☒	The statistical test(s) used AND whether they are one- or two-sided<br>*Only common tests should be described solely by name; describe more complex techniques in the Methods section.*
☐	☒	A description of all covariates tested
☐	☒	A description of any assumptions or corrections, such as tests of normality and adjustment for multiple comparisons
☐	☒	A full description of the statistical parameters including central tendency (e.g. means) or other basic estimates (e.g. regression coefficient) AND variation (e.g. standard deviation) or associated estimates of uncertainty (e.g. confidence intervals)
☐	☒	For null hypothesis testing, the test statistic (e.g. F, t, r) with confidence intervals, effect sizes, degrees of freedom and P value noted<br>*Give P values as exact values whenever suitable.*
☒	☐	For Bayesian analysis, information on the choice of priors and Markov chain Monte Carlo settings
☒	☐	For hierarchical and complex designs, identification of the appropriate level for tests and full reporting of outcomes
☒	☐	Estimates of effect sizes (e.g. Cohen's d, Pearson's r), indicating how they were calculated

Our web collection on statistics for biologists contains articles on many of the points above.

Software and code

Policy information about availability of computer code

Data collection	Mass spectrometry data was collected with software linked to the Q Exactive Plus Hybrid Quadrupole-Orbitrap instrument (Thermo Fisher).
Data analysis	LC-MS data was searched using the SEQUEST search engine within the Proteome Discoverer software platform, version 1.4 (Thermo Fisher), using the SwissProt human database that was downloaded in December of 2015.<br>Image analysis of immunoblots were performed using Image Studio software (Li-Cor).<br>Image analysis of Phosphorimager scans were performed using ImageJ open source software.<br>All statistics and graphs were performed and generated using GraphPad Prism software.

For manuscripts utilizing custom algorithms or software that are central to the research but not yet described in published literature, software must be made available to editors/reviewers. We strongly encourage code deposition in a community repository (e.g. GitHub). See the Nature Research guidelines for submitting code & software for further information.

Data

Policy information about availability of data

All manuscripts must include a data availability statement. This statement should provide the following information, where applicable:

- Accession codes, unique identifiers, or web links for publicly available datasets
- A list of figures that have associated raw data
- A description of any restrictions on data availability

The authors declare that all data supporting the findings of this study are available within the paper and its supplementary information files.

Field-specific reporting

Please select the one below that is the best fit for your research. If you are not sure, read the appropriate sections before making your selection.

☒ Life sciences ☐ Behavioural & social sciences ☐ Ecological, evolutionary & environmental sciences

For a reference copy of the document with all sections, see nature.com/documents/nr-reporting-summary-flat.pdf

Life sciences study design

All studies must disclose on these points even when the disclosure is negative.

Sample size	For each study, the sample size was chosen to ensure reproducibility between independent experiments. The statistics were then analyzed using Graphpad Software to provide confidence in any statistics based upon the statistical methods used.
Data exclusions	No data was excluded.
Replication	All experiments were replicated.
Randomization	No randomization was performed
Blinding	No blinding was performed since no subjective measures were part of the analysis.

Reporting for specific materials, systems and methods

We require information from authors about some types of materials, experimental systems and methods used in many studies. Here, indicate whether each material, system or method listed is relevant to your study. If you are not sure if a list item applies to your research, read the appropriate section before selecting a response.

Materials & experimental systems

n/a	Involved in the study	
☐	☒	Antibodies
☐	☒	Eukaryotic cell lines
☒	☐	Palaeontology
☒	☐	Animals and other organisms
☐	☒	Human research participants
☒	☐	Clinical data

Methods

n/a	Involved in the study	
☒	☐	ChIP-seq
☒	☐	Flow cytometry
☒	☐	MRI-based neuroimaging

Antibodies

Antibodies used	Proteintech DALRD3 (26294-1-AP)<br>Sigma-Aldrich Anti-FLAG M2 (F1804)<br>GenScript anti-twinStrep THETM NWSHPQFEK antibody (A01732)<br>Santa Cruz, anti-GFP, cat. No. sc-9996<br>EMD Millipore, anti-Actin C4 (cat. No MAB1501)
Validation	Proteintech DALRD3: Validated by manufacturers (https://www.ptglab.com/products/DALRD3-Antibody-26294-1-AP.htm#validation) and in our manuscript (See Figures 5 and 6)<br>Sigma-Aldrich Anti Flag M2: Validated by manufacturers (https://www.sigmaaldrich.com/catalog/product/sigma/f1804?lang=en&region=US&gclid=Cj0KCQjw_OzrBRDmARIsAAIdQ_Lk5s4Y5qR2sCiCyqQBnHG9fL3OriUs0ExQUI8gpoJNxg1INrCcLw4aAoE0EALw_wcB) and in our manuscript (See Figures 2, 3 and 5)<br>GenScript anti-twin-Strep THETM NWSHPQFEK: Validated by the manufacturers (https://www.genscript.com/antibody/A01732-THE_sup_TM_sup_NWSHPQFEK_Tag_Antibody_mAb_Mouse.html) and in our manuscript (Figures 1, 2 and 3)<br>Santa Cruz anti-GFP, https://www.scbt.com/p/gfp-antibody-b-2<br>EMD Millipore, anti-actin C4, https://www.sigmaaldrich.com/catalog/product/mm/mab1501?lang=en&region=US

Eukaryotic cell lines

Policy information about cell lines

Cell line source(s)	HAP1: Horizon Discovery, Human Male Chronic Myelogenous Leukemia (CML) cell line KBM-7<br>HEK-293T: ATCC, Human Embryonic Kidney
Authentication	The original HAP1 cells used to generate the cell lines in this study have been extensively characterized by whole genome

Authentication	sequencing and transcriptome analysis. The subsequent HAP1 knockout cell lines were validated by PCR amplification and Sanger Sequencing to confirm the mutation at the genomic level. The 293T cell lines were obtained from ATCC which validates all cell lines using STR profiling.
Mycoplasma contamination	Cell lines were negative for mycoplasma contamination as tested by Horizon Discovery and subsequently by our lab.
Commonly misidentified lines (See ICLAC register)	None

Human research participants

Policy information about studies involving human research participants

Population characteristics	Individuals with a documented intelligent quotient of 70 or less were eligible for the study. Younger children (<5 years) were eligible if developmental assessment by a pediatric neurologist revealed delayed acquisition of speech and other cognitive developmental domains regardless of whether other developmental domains were also involved (cases were labeled as developmental delay or global developmental delay accordingly). All subjects were evaluated by board-certified neurologists and clinical geneticists.
Recruitment	All patients with Mendelian phenotypes are eligible. Participants were recruited from a Saudi nation-wide screen for patients with unidentified neurodevelopmental disorders as referenced in Anazi et al. 2017. There is self-selection bias since families with neurodevelopmental disorders were selected or volunteered for the study. This does not impact the results since all prospective genetic loci and molecular phenotypes are monitored and validated in this study.
Ethics oversight	KFSHRC IRB RAC# 2121053

Note that full information on the approval of the study protocol must also be provided in the manuscript.
