# Supplementary material for: DALRD3 encodes a protein mutated in epileptic encephalopathy that targets arginine tRNAs for 3-methylcytosine modification
Source: Nat Commun. 2020 May 19;11:2510. doi: 10.1038/s41467-020-16321-6 (PMC7237682; doi:10.1038/s41467-020-16321-6)
Supplement: Supplementary file 4 — Description of Additional Supplementary Files [file 41467_2020_16321_MOESM4_ESM.pdf]

Description of Additional Supplementary Files

File Name: Supplementary Data 1
Description: Protein identification and peptide matches from liquid chromatographymass spectrometry analysis of METTL2A, METTL2B and METTL6 purifications.

File Name: Supplementary Data 2
Description: Protein identification and peptide matches from liquid chromatographymass spectrometry analysis of DALRD3 purification.
